# Mapping Evidence on the Burden of Breast, Cervical, and Prostate Cancers in Sub-Saharan Africa: A Scoping Review

**DOI:** 10.3389/fpubh.2022.908302

**Published:** 2022-06-16

**Authors:** Alfred Musekiwa, Maureen Moyo, Mohanad Mohammed, Zvifadzo Matsena-Zingoni, Halima Sumayya Twabi, Jesca Mercy Batidzirai, Geoffrey Chiyuzga Singini, Kabelo Kgarosi, Nobuhle Mchunu, Portia Nevhungoni, Patricia Silinda, Theodora Ekwomadu, Innocent Maposa

**Affiliations:** ^1^School of Health Systems and Public Health, Faculty of Health Sciences, University of Pretoria, Pretoria, South Africa; ^2^School of Mathematics, Statistics, and Computer Science, University of KwaZulu-Natal, Pietermaritzburg, South Africa; ^3^Division of Epidemiology and Biostatistics, School of Public Health, University of the Witwatersrand, Johannesburg, South Africa; ^4^Department of Mathematical Sciences, University of Malawi, Zomba, Malawi; ^5^Biostatistics Unit, South African Medical Research Council, Durban, South Africa; ^6^Biostatistics Unit, South African Medical Research Council, Pretoria, South Africa; ^7^Department of Biological Sciences, Faculty of Natural and Agricultural Sciences, North-West University, Mmabatho, South Africa

**Keywords:** breast cancer, cervical cancer, prostate cancer, burden, Sub-Saharan Africa

## Abstract

**Background:**

Cancer remains a major public health problem, especially in Sub-Saharan Africa (SSA) where the provision of health care is poor. This scoping review mapped evidence in the literature regarding the burden of cervical, breast and prostate cancers in SSA.

**Methods:**

We conducted this scoping review using the Arksey and O'Malley framework, with five steps: identifying the research question; searching for relevant studies; selecting studies; charting the data; and collating, summarizing, and reporting the data. We performed all the steps independently and resolved disagreements through discussion. We used Endnote software to manage references and the Rayyan software to screen studies.

**Results:**

We found 138 studies that met our inclusion criteria from 2,751 studies identified through the electronic databases. The majority were retrospective studies of mostly registries and patient files (*n* = 77, 55.8%), followed by cross-sectional studies (*n* = 51, 36.9%). We included studies published from 1990 to 2021, with a sharp increase from 2010 to 2021. The quality of studies was overall satisfactory. Most studies were done in South Africa (*n* = 20) and Nigeria (*n* = 17). The majority were on cervical cancer (*n* = 93, 67.4%), followed by breast cancer (67, 48.6%) and the least were on prostate cancer (48, 34.8%). Concerning the burden of cancer, most reported prevalence and incidence. We also found a few studies investigating mortality, disability-adjusted life years (DALYs), and years of life lost (YLL).

**Conclusions:**

We found many retrospective record review cross-sectional studies, mainly in South Africa and Nigeria, reporting the prevalence and incidence of cervical, breast and prostate cancer in SSA. There were a few systematic and scoping reviews. There is a scarcity of cervical, breast and prostate cancer burden studies in several SSA countries. The findings in this study can inform policy on improving the public health systems and therefore reduce cancer incidence and mortality in SSA.

## Introduction

The burden of cancer is on a rise in developing countries due to the demographic transitions and changes in life-style behaviors (1–4). The most common cancer cases among women in Sub-Saharan Africa (SSA) are breast (25% of all cancers) and cervical (24% of all cancers), while prostate cancer (23% of all cancers) is common among men in SSA (5, 6). Of all the 2018 cancer deaths among women in SSA, 21.7% were attributed to cervical cancer, making it the most common cause of cancer death in the region (7). Breast cancer mortality is high in SSA compared to developed countries (8–13). The prevalence of prostate cancer among men has been high in SSA as it is the leading cause of cancer deaths, particularly among men of African origins (14).

Determining the burden of prostate cancer within SSA has been challenging due to the lack of reliable reporting. The incidence of breast cancer has been reported to be higher in rural areas (60–75%) than in urban areas in SSA (15), and many of these women go untreated, mostly due to a lack of access to health care. One study found that prostate cancer is a serious challenge in African men and its actual incidence is underestimated in SSA settings due to poor access to healthcare, lack of screening, genetic predisposition, lifestyle and environmental factors (4). According to Jalloh et al. (16), most of the health interventions in SSA have focused on communicable diseases such as malaria, tuberculosis and HIV, however, cancer care has been given limited attention.

This study aims to determine recent trends in the burden, including incidence, prevalence, and disability-adjusted life years (DALYs), of breast, cervical and prostate cancer in SSA. Highlighting the burden of the three most common cancers helps bring disparate information reported in the literature to policy makers in an easy and accessible format, in one place. The focus is on SSA because this is where early marriages are rampant and hence girls younger than 15 years old are exposed to early sexual activity, thus increasing their risk of contracting Human Papilloma Virus (HPV), a risk factor for cervical cancer, at a young age (17). In addition, evidence has shown that cervical cancer is more prevalent in HIV-infected women compared to the HIV-uninfected, and their survival rates are poorer (18). Furthermore, breast and prostate cancer awareness and treatment are lower in SSA due to limited resources. This scoping review seeks to provide evidence on the burden of these three most common types of cancers in SSA. Results from this study will add to the body of literature and help policy makers in making informed decisions that will improve the public health systems in reducing the cancer burden in SSA.

## Methods

### Study Design

This scoping review aimed to collate, synthesize, and analyze the wide range of available evidence to map literature on the burden of breast, cervical and prostate cancers in SSA. The title was registered with the Open Science Framework (OSF) (https://archive.org/details/osf-registrations-bna26-v1). The scoping review used the Arksey and O'Malley framework (19) as reviewed and updated by Levac et al. (20) and the Joanna Briggs Institute 2020 guidelines (21). The framework has the following five steps that guide how scoping reviews are done:

Step 1: Identifying the research question.
Step 2: Identifying relevant studies.
Step 3: Selecting studies
Step 4: Charting the data.
Step 5: Collating, summarizing and reporting the data.

The scoping review was reported using the Preferred Reporting Items for Systematic Reviews and Meta-analysis: Extension for Scoping Reviews (PRISMA-ScR) guidelines (22).

#### Identifying the Research Question

Our primary research question for the scoping review was: What evidence exists on the epidemiological burden of breast, cervical and prostate cancers in SSA? The measures of burden include spatial distribution, incidence, prevalence, quality-adjusted life years (QALYs), disability-adjusted life years (DALYs), and years of life lost (YLL).

To assess the eligibility of the research question, this scoping review utilized the Population, Concept and Context (PCC) mnemonic, derived by the Joanna Briggs Institute (21). The PCC statement guided the selection of studies (Table 1).

**Table 1:** PCC framework used to determine the eligibility of the research question and to guide the selection of studies on the burden of breast, cervical and prostate cancers.

**Population**	**Adult cancer patients (breast, cervical, prostate)**
Concept	Burden of breast, cervical, and prostate cancer
Context	Sub-Saharan Africa

#### Identifying Relevant Studies

We conducted a comprehensive search from inception to 25 October 2021, irrespective of language, in the following electronic databases: PubMed, Web of Science, and Scopus. The search strategy was developed with an experienced librarian (KK, who is also the co-author) using a variety of keywords related to the burden of breast, cervical and prostate cancers in SSA. A combination of these keywords and Medical Subject Heading (MeSH) terms were used and tailored appropriately to each database. In addition, we manually searched the reference lists of all included studies to identify additional literature. Table 2 shows a pilot search strategy used in PubMed. The actual search strategies used are given in the Supplementary Materials.

**Table 2:** PubMed pilot search strategy for SSA studies on cervical, breast and prostate cancer.

**Date**	**Database**	**Keywords**
25/10/2021	PubMed	Search: ((((“prostatic neoplasms”[MeSH Terms] OR Prostate cancer[Text Word] OR prostatic neoplasm*[Text Word] OR prostate tumour*[Text Word]) OR (“uterine cervical neoplasms”[MeSH Terms] OR Cervical cancer[Text Word] OR Cervix Cancer[Text Word] OR uterine cervical neoplasms[Text Word] OR cervical neoplasms[Text Word])) OR (“breast neoplasms”[MeSH Terms] OR Breast Cancer[Text Word] OR breast neoplasm[Text Word] OR Breast Tumor*[Text Word])) AND (“Global Burden of Disease”[MeSH Terms] OR burden[Text Word] OR “prevalence”[MeSH Terms] OR prevalence[Text Word])) AND ((“africa south of the sahara”[MeSH Terms] OR Sub saharan Africa[Text Word] OR SSA[Text Word] OR Sub-saharan Africa[Text Word] OR Sub sahara Africa[Text Word]) OR ((“Africa”[MeSH Terms] OR AFRICA[Text Word]) NOT (“Africa, Northern”[Mesh] OR Northern Africa[Text Word])))

##### Eligibility Criteria

We conducted an extensive title and abstract screening guided by the study's eligibility criteria to determine all eligible articles to be selected for this scoping review. To account for all eligible studies included in the review, the inclusion and exclusion criteria listed below were followed.

*Inclusion Criteria*

This study included all publications that adhered to the following criteria:

- Studies reporting on at least one of the types of cancers, that is, breast, cervical and prostate.
- Studies reporting on the burden (prevalence, incidence, QALYs, DALYs, and others) on at least one of breast, cervical and prostate cancers.
- All publications reporting evidence on the three cancers in different SSA countries; this was not limited by study design.
- Studies from all countries in SSA (all countries in Africa except Algeria, Egypt, Morocco, Tunisia, and Libya).
- Studies from inception to search date.

*Exclusion Criteria*

This review excluded studies based on the following:

- Any articles reporting on cancers other than cervical, breast and prostate cancers
- Northern African countries (Algeria, Egypt, Morocco, Tunisia, and Libya) which are not considered SSA.

#### Selecting Studies

We screened the titles of the associated selected articles from the different repositories. After that, we used EndNote 20 software to identify the duplicates in the qualified articles and deleted them. The abstracts of the qualified articles were examined by two independent reviewers using a screening tool that specifies inclusion and exclusion criteria. In this stage, the reviewers resolved any contradiction by involving a third reviewer. The two reviewers then explored the full text of the selected articles using the screening tool. After this phase, any contradictions arising were resolved independently by a third reviewer. For any articles that could not be accessed during the screening phase, the librarian assisted in retrieving the full-text articles. The database searches, keywords used, and the number of selected articles were properly reported. Also, the screening results were displayed using a PRISMA-ScR flow diagram (22).

#### Charting the Data

To extract relevant information from each of the selected articles, an electronic data charting form was developed with data extraction tool components, including author and date, title, country, study design, type of cancer (cervical, breast, prostate), type of burden reported, and key findings. The data extraction tool was piloted by two independent reviewers before use. Any appropriate changes were then applied as per the feedback received during the piloting stage and the tool was updated accordingly.

##### Quality Appraisal

We used the Mixed Methods Appraisal Tool (MMAT) V.2018 software (23) to assess the quality of studies since we anticipated different types of study designs to be included in the scoping review. The tool included the relevance of the study, the study design, adequacy and methodology, data collection, data analysis and the main study findings. The process of quality appraisal was performed independently and in duplicate by at least two review authors to avoid any bias. Disagreements between reviewers were resolved through discussion.

#### Collating, Summarizing and Reporting Results

We summarized the emerging themes for the burden of the three cancers using a narrative approach. The summary was structured around the burden and types of cancers. We also meticulously analyzed and reported any other emerging themes relevant to the research question. Where appropriate we used tables and graphs to visualize findings. The results of this scoping review were used to determine the gaps in knowledge regarding the burden of breast, cervical and prostate cancer in SSA.

## Results

### Results of the Search

**Figure 1:**
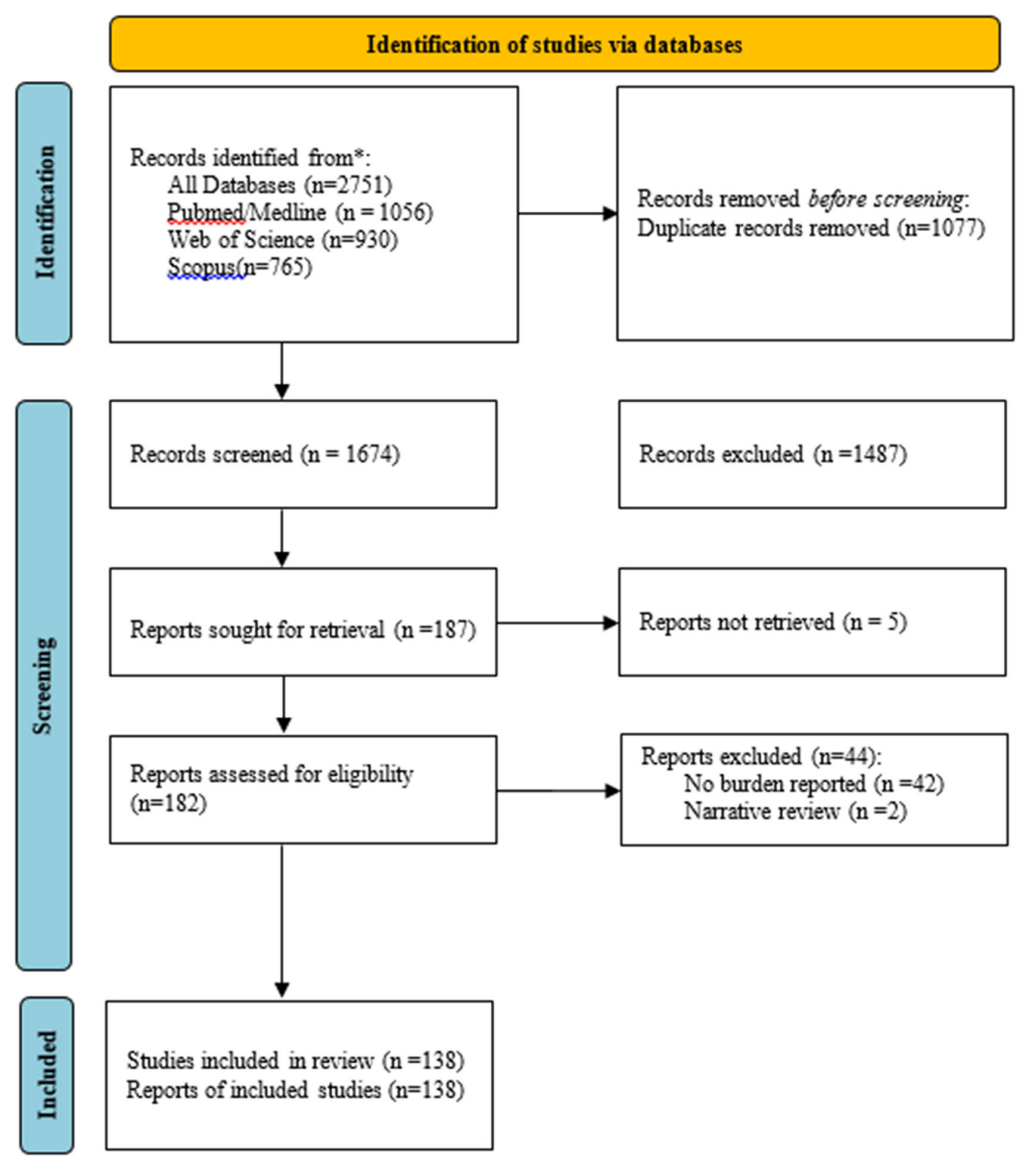
PRISMA flow chart showing the number of studies included in a scoping review for breast, cervical and prostate cancer in Sub-Saharan Africa.

A total of 2,751 studies were found in three electronic databases (PubMed, Web of Science, and Scopus). After removing 1,077 duplicate records, we remained with 1,674 records whose titles and abstracts were screened. Screening resulted in 1,487 records being excluded for being irrelevant, thus leaving 187 reports that were sought to have their full texts retrieved, however, five records could not be retrieved, and this resulted in 182 full-text reports that were assessed for eligibility by at least two reviewers. Finally, 44 records were excluded after the full-text screening, and therefore 138 studies were included in this scoping review (Figure 1).

### Characteristics of Included Studies

#### Study Designs and Quality of Included Studies

##### Study Designs

**Figure 2:**
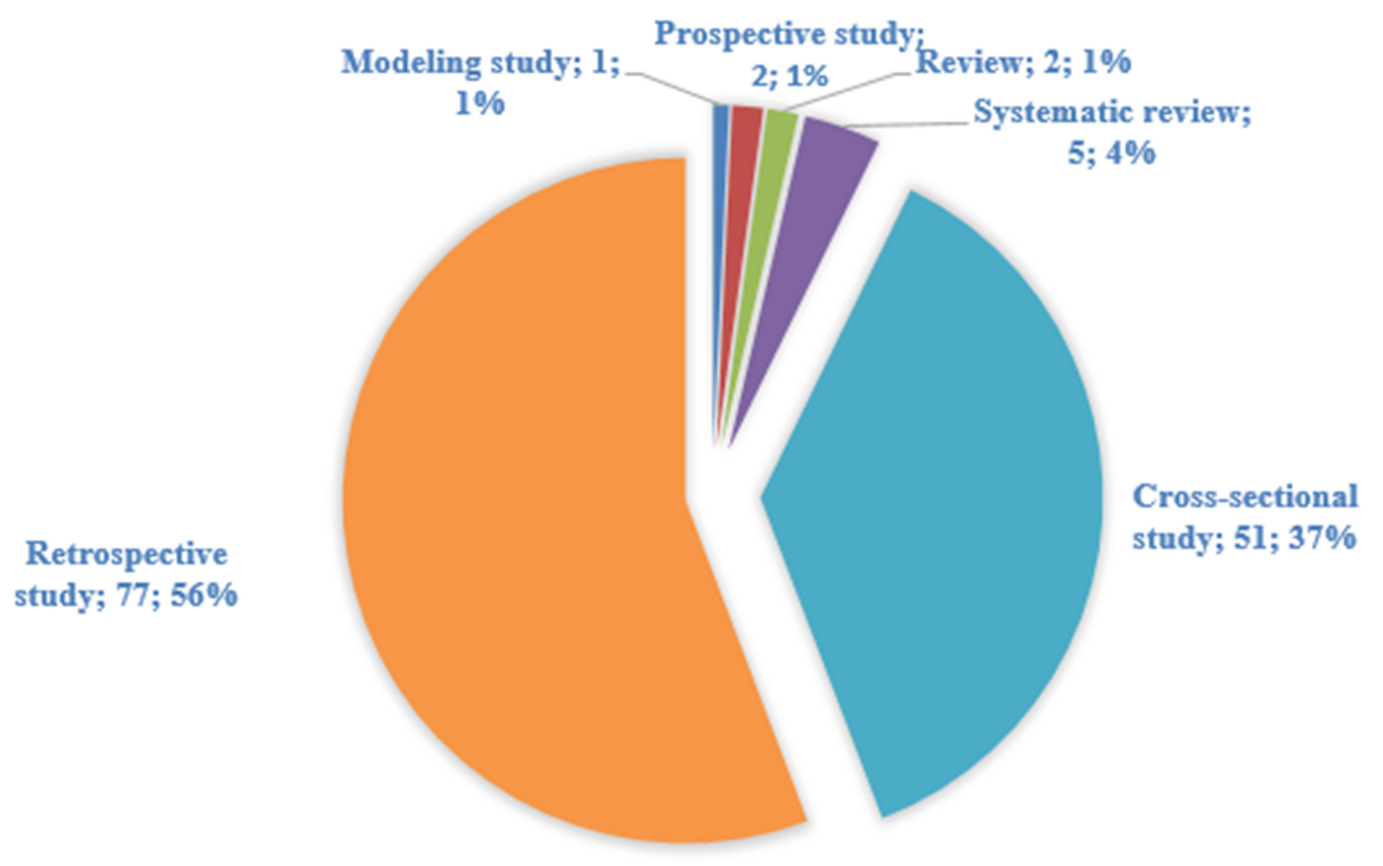
Pie chart showing study designs for studies on breast, cervical and prostate cancer in Sub-Saharan Africa.

The majority of the included studies were retrospective studies (*n* = 77, 55.8%) of mostly registries and patient files (6, 10, 24–98) followed by cross-sectional studies (*n* = 51, 36.9%) (99–149), systematic reviews (*n* = 5, 3.6%) (2, 150–153) and reviews (*n* = 2, 2.1%) (154, 155). There were two prospective studies (156, 157) and one modeling study (158) (Figure 2).

The prevalence studies had different target populations, with some only targeting cancer patients and calculating the percentage of each type of cancer. Some cross-sectional studies were targeting the general population, including screening studies.

##### Year of Publication

**Figure 3:**
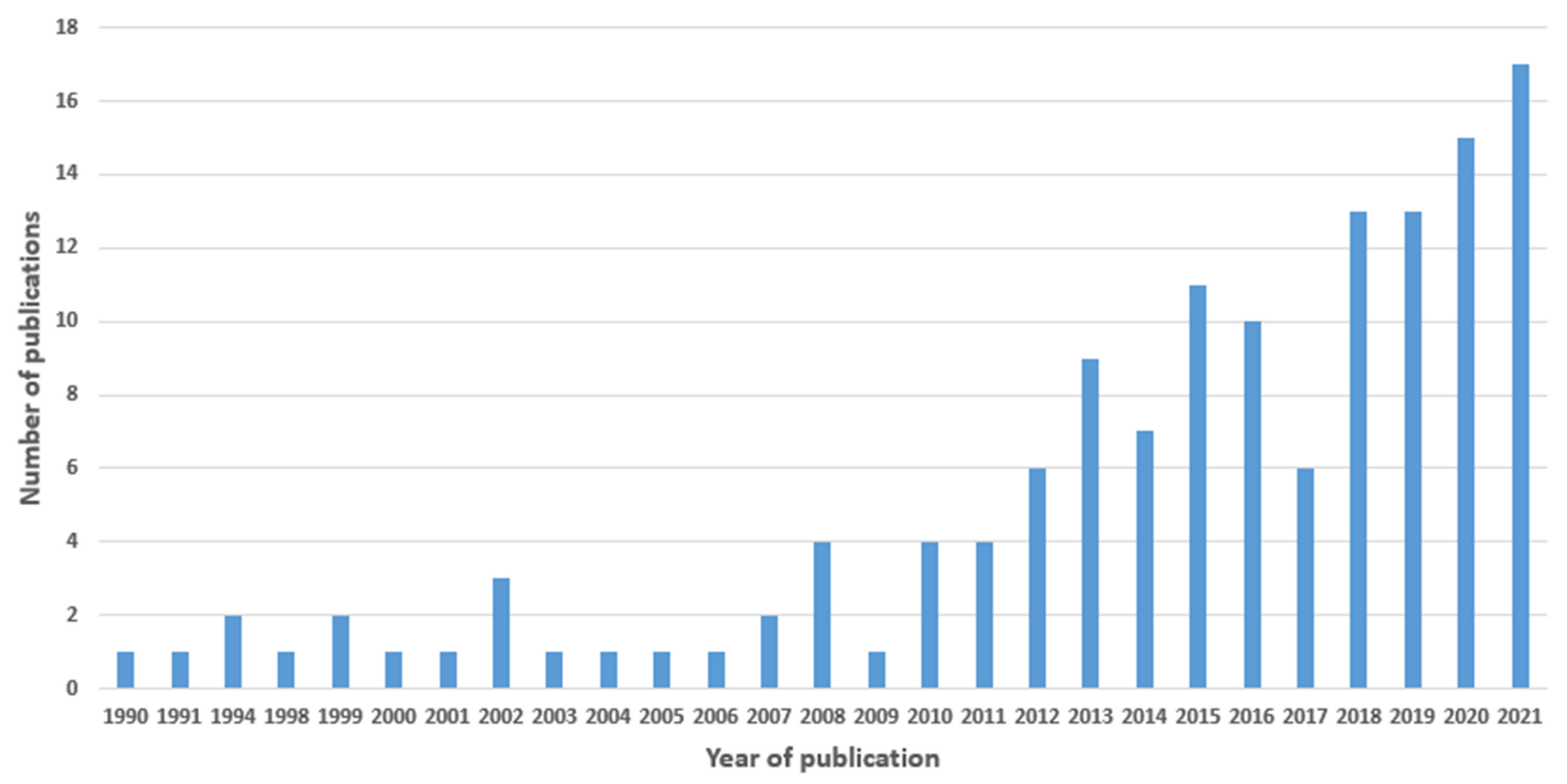
Trend in the number of included studies over time in years, 1990 to 2021.

We included studies published from 1990 to 2021, with a sharp increase from 2010 to 2021 (Figure 3).

##### Quality of Studies (MMAT Tool)

The quality of studies, assessed using the Mixed Methods Assessment Tool (MMAT), is summarized in Table 3. The assessment excludes the five systematic reviews, two reviews, and the modeling study. Therefore, the assessment was carried out for 130 studies; the majority were quantitative studies (*n* = 129) and they satisfied all the qualities in the table; appropriate sampling strategy, representative sample, appropriate measurements, low nonresponse bias, and using appropriate statistical methods. There was one mixed-methods study (133), which had an adequate rationale for mixed methods and had both the quantitative and qualitative aspects adequately interpreted.

**Table 3:** MMAT table for Sub-Saharan African studies on the burden of cervical, breast and prostate cancer.

**Study type**	**Question**	**Yes**	**No**	**Cannot tell**	**Total**
SCREENING QUESTIONS	S1. Are there clear research questions?	128	0	2	130
	S2. Do the collected data allow us to address the research questions?	129	0	1	130
	4.1. Is the sampling strategy relevant in addressing the research question?	127	1	1	129
	4.2. Is the sample representative of the target population?	126	1	2	129
QUANTITATIVE DESCRIPTIVE STUDIES	4.3. Are the measurements appropriate?	127	1	1	129
	4.4. Is the risk of nonresponse bias low?	70	24	35	129
	4.5. Is the statistical analysis appropriate to answer the research question?	125	1	3	129
	5.1. Is there an adequate rationale for using a mixed-methods design to address the research question?	1	0	0	1
	5.2. Are the different components of the study effectively integrated to answer the research question?	0	1	0	1
MIXED METHODSSTUDIES	5.3. Are the outputs of the integration of qualitative and quantitative components adequately interpreted?	1	0	0	1
	5.4. Are divergences and inconsistencies between quantitative and qualitative results adequately addressed?	0	1	0	1
	5.5. Do the different components of the study adhere to the quality criteria of each tradition of the methods involved?	0	0	1	1

#### Spatial Distribution of Studies

**Figure 4:**
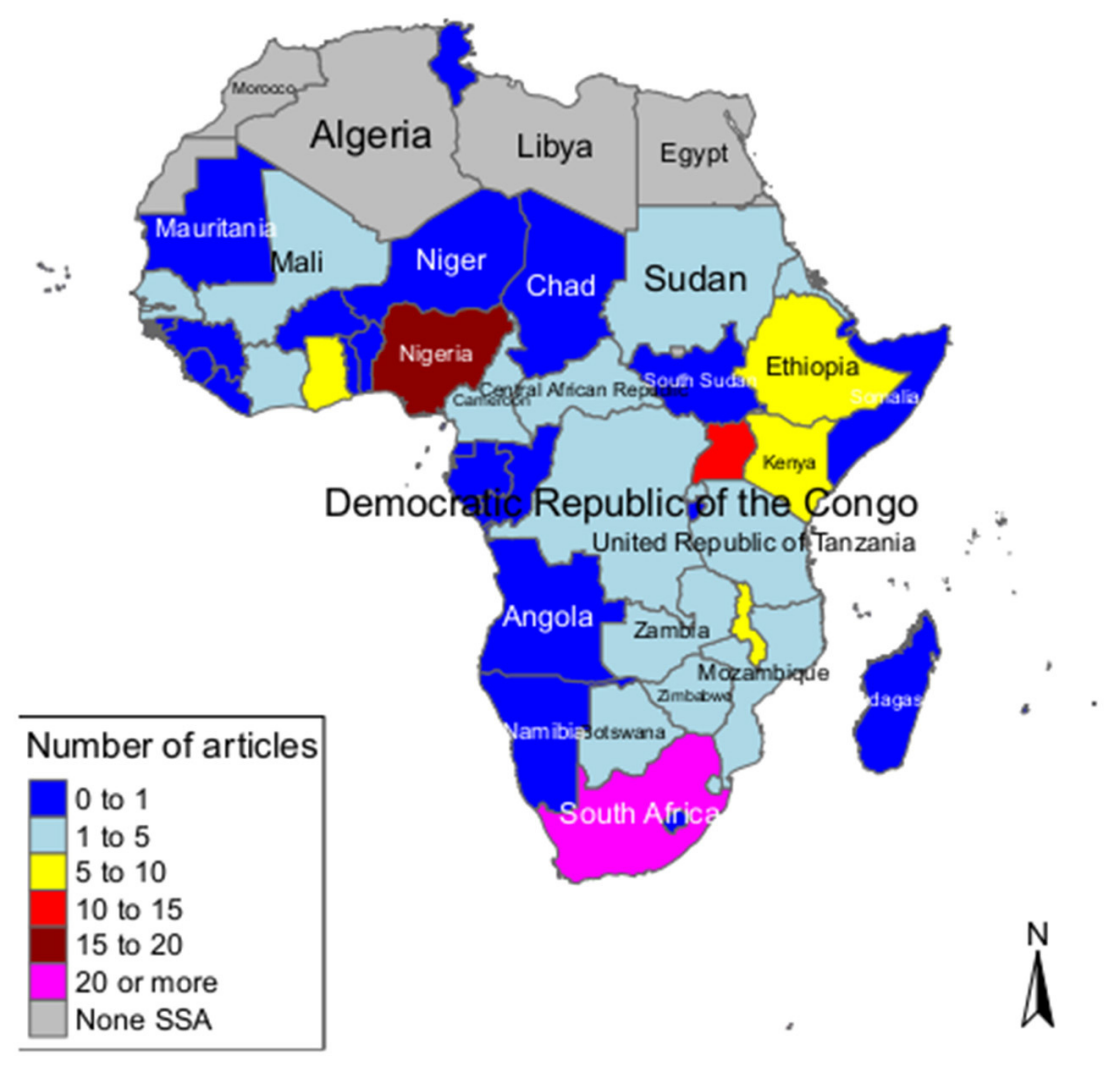
Map showing spatial distribution of studies on breast, cervical and prostate cancer in Sub-Saharan Africa (global and multi-country studies were not included on the map).

While most studies were single-country studies, some were multi-country and global studies including African countries. There were 16 global studies including the African or SSA regions (27, 46, 69, 104, 106, 108, 109, 117, 121, 122, 127, 131, 142, 144, 145, 158) and there were 16 African multi-country studies (2, 6, 87, 119, 120, 138, 140, 141, 143, 148–154). From the single-country studies, majority were done in Nigeria (*n* = 17) (24, 25, 28, 29, 34, 45, 54, 64, 72, 75, 92, 93, 103, 113, 116, 124, 137) and South Africa (*n* = 20) (10, 26, 36, 42, 55, 65, 80–83, 86, 88, 89, 91, 98, 105, 110, 125, 129, 135), followed by Uganda (*n* = 10) (35, 40, 48, 59, 74, 78, 79, 90, 94, 95), then Malawi (*n* = 9) (37, 52, 53, 56, 66–68, 118, 133) and Ethiopia (*n* = 9) (33, 51, 63, 97, 101, 107, 112, 146, 157). We also found studies conducted in Ghana (*n* = 6) (50, 71, 111, 123, 134, 155), Kenya (*n* = 5) (70, 96, 115, 132, 139), Zambia (*n* = 4) (44, 77, 102, 126), and Eritrea (*n* = 3) (60–62). There were seven countries with two studies each [Botswana (43, 47), the Central African Republic (31, 32), Mozambique (57, 58), Rwanda (130, 136), Senegal (147, 156), Sudan (84, 114) and Zimbabwe (38, 39)]. The countries with a single study were Cote d'Ivoire (128), Democratic Republic of Congo (99), Mali (85), Swaziland (76), Tanzania (49), and The Gambia (30). We found no studies in the following SSA countries: Angola, Benin, Burkina Faso, Burundi, Cape Verde, Comoros, Equatorial Guinea, Gabon, Guinea, Guinea-Bissau, Lesotho, Liberia, Madagascar, Mauritania, Mauritius, Namibia, Niger, Sao Tome, Seychelles, Sierra Leone, Somalia, and Togo (Figure 4).

#### Types of Cancer

**Figure 5:**
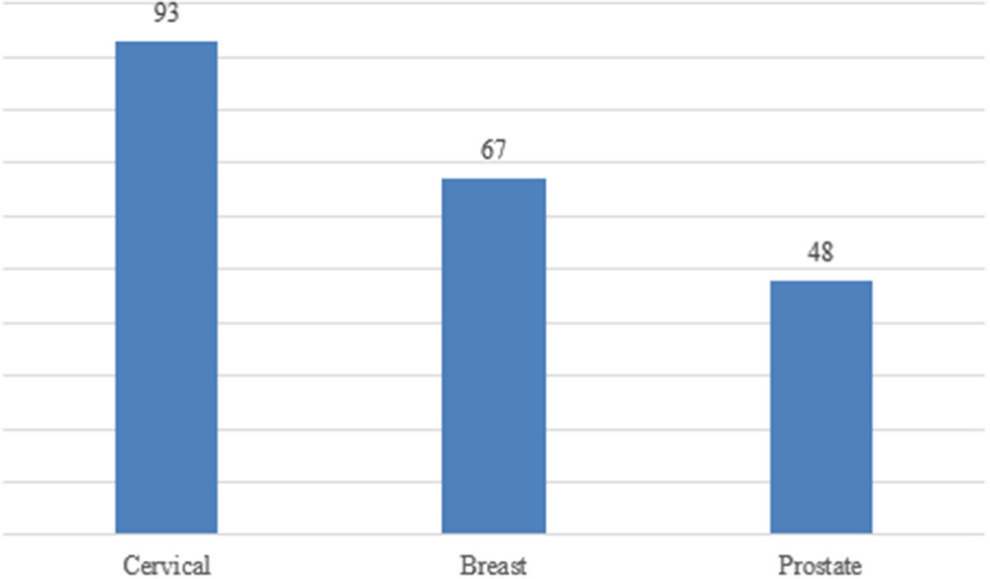
Figure showing the distribution of studies on breast, cervical and prostate cancer in Sub-Saharan Africa (some studies included more than one type of cancer).

The majority of the studies were on cervical cancer (*n* = 93, 67.4%), followed by breast cancer (67, 48.6%) and the least were on prostate cancer (48, 34.8%); some studies investigated all the three types of cancer (*n* = 29) while some reported two of the three cancers (*n* = 15) (Figure 5).

### Burden of Cancer

#### Cervical Cancer

##### Prevalence Studies

A total of 51 studies reported the prevalence of cervical cancer in Sub-Saharan Africa, of which the majority (*n* = 9, 17.6%) were from Nigeria (28, 34, 45, 54, 64, 92, 93, 113, 137); these were retrospective record reviews of registries and patient files and two were cross-sectional studies (113, 137). This was followed by six studies from Malawi: one cross-sectional study (118), one cross-sectional study of the Malawi Cancer Registry (133), two retrospective cohort studies (66, 68) and two retrospective record reviews of registries (53, 67). There were four studies from South Africa: a cross-sectional study (105), a retrospective cohort study (55), and two retrospective record reviews; registry (91) and patient files (65). There were also four studies from Uganda, which were retrospective record reviews of registries (59, 79, 94) and pathology reports (95). There were also three studies from Kenya: one cross-sectional study (132) and two retrospective record reviews of patient files (70) and registry (96). Another three studies were from Zambia: two cross-sectional studies (102, 126) and one retrospective record review of patient files (77). There were also three studies from Ethiopia: two cross-sectional studies (112, 146) and one retrospective review of patient files (51). There were two studies from Cameroon; both retrospective record reviews of patient files (41) and registry (73). One cross-sectional study was conducted in each of the following countries: Côte d'Ivoire (128), the Democratic Republic of Congo (99), Ghana (111), Rwanda (130), and Senegal (147). There was also one retrospective record review of patient files from Botswana (47) and another retrospective record review of smears from Swaziland (now called Eswatini) (76).

Six studies were African multi-country studies: two African studies; one cross-sectional study of Global Cancer Incidence, Mortality and Prevalence (GLOBOCAN), an online database providing global cancer statistics and estimates of incidence and mortality in 185 countries for 36 types of cancer, and all cancer sites combined (138) and a scoping review study (154), one cross-sectional study from South Africa and Uganda (120), and three systematic reviews [one from SSA (151), one from seven African countries (152) and one from Southern and Eastern Africa (153)]. There were three GLOBOCAN studies (104, 127, 145).

##### Incidence Studies

A total of 36 studies looked at the incidence of cervical cancer in Sub-Saharan Africa, of which the majority were from South Africa: two retrospective reviews of registries (10, 83), two retrospective cohort studies (81, 82), one retrospective record review of pathology reports (86) and one cross-sectional study (135). There were five studies from Uganda consisting of three record reviews of registries (35, 59, 74), one retrospective cohort study (40), and one retrospective record review of pathology reports (78). Three retrospective record reviews of registries were from Malawi (37, 52, 67). Two studies were retrospective record reviews of registries from Nigeria (24, 75). Two retrospective record reviews of registries from Zimbabwe (38, 39). Two Ethiopian retrospective record reviews of patient files (51) and registry (63) and two retrospective record reviews from Mozambique; one using pathology reports (57) and the other registry data (58).

There were five African multi-country studies: one systematic review including seven African countries (152) and four SSA studies; three cross-sectional studies (140, 141, 149) and one retrospective review of registry (6). There were five global studies, of which two were cross-sectional GLOBOCAN studies (109, 121), one cross-sectional study of Global Burden of Disease Study (117), a retrospective record review of registry (27), and a modeling study (158).

There was one study from each of the following countries: one retrospective record review of registry from The Gambia (30), one retrospective review of pathology reports from Eritrea (61), Ghana (71) and Sudan (84).

##### Other Burden Measures

A total of 11 studies considered other measures of cervical cancer burden; five studies reported mortality globally including Africa (117, 144), South Africa (129), Kenya (139) and Ethiopia (97). One cross-sectional study reported the population attributable fraction in a GLOBOCAN study (108) Two cross-sectional studies reported the disability-adjusted life years (DALYs) in Kenya (115) and for the SSA region (119). One GLOBOCAN study reported years of life lost (YLL) (148). Two cross-sectional studies in South Africa reported odds ratios for cervical cancer comparing HIV infected with HIV uninfected individuals (42, 110).

#### Breast Cancer

##### Prevalence Studies

A total of 32 studies reported the prevalence of breast cancer in Sub-Saharan Africa. Five studies were from Nigeria, of which four were retrospective record reviews of registries (28, 45, 64, 72) and one was a cross-sectional study (116). Four studies were from Ethiopia, three cross-sectional studies (101, 107, 146) and one retrospective review of patient files (51). Three studies were from Malawi, where one was a retrospective review of the registry (53) and two were retrospective cohort studies (66, 68). There were three studies from Uganda, two were retrospective record reviews of pathology reports (90, 95) and one was a retrospective record review of registry (59). Two studies were from South Africa, one retrospective record review (26) and a cross-sectional study of Demographic and Health Survey (DHS) data (125). Two studies were from Ghana, of which one was a cross-sectional study (134) and the other a retrospective record review of patient files (50). There was one study from each of the following countries: a retrospective record review of pathology reports from the Central African Republic (32), another retrospective record review of non-governmental records in Botswana (43), one cross-sectional study from Sudan (114), one cross-sectional study in both South Africa and Uganda (120), one retrospective record review of registry from Kenya (96), and a retrospective record review of pathology reports in Zambia (44).

There were two studies covering the whole of Africa, one a scoping review (154) and the other a GLOBOCAN cross-sectional study (138). There were also two GLOBOCAN cross-sectional studies (104, 145).

##### Incidence Studies

There were 28 studies reporting the incidence of breast cancer in Sub-Saharan Africa. Ten of the studies were covering the whole of Africa or SSA; a systematic review (2), three cross-sectional GLOBOCAN studies (122, 131, 143), three cross-sectional studies of the WHO database (106), Global Burden of Disease Study (117) and registries (141), two retrospective record review of registries (87, 117) and a retrospective cohort study (69). In South Africa, there were three incidence studies on breast cancer; two retrospective record reviews of registries (89) and mammograms (98) and a cross-sectional study of the Statistics South Africa registry (135). There were also three studies from Uganda; one retrospective cohort study (40), and retrospective record review of registry (35) and pathology reports (78).

There were two Ethiopian studies on the incidence of breast cancer; one prospective observational study (157) and a retrospective record review of a registry (63). Also, two incidence studies in Mozambique consisted of a retrospective record review of pathology reports (57) and registry (58).

There was one incidence study from each of the following countries: a retrospective record review of registry in Nigeria (24), The Gambia (30), the Central African Republic (31), Eritrea (62), Malawi (37), Zimbabwe (38), Mali (85), and Sudan (84).

##### Other Burden Measures

A total of 12 studies considered other measures of breast cancer burden; mortality was measured by nine studies (46, 48, 69, 98, 117, 129, 139, 143, 144). DALYs were reported by four incidence studies (46, 69, 117, 119). One study reported quality of life (156) and another reported economic burden (142).

#### Prostate Cancer

##### Prevalence Studies

A total of 23 studies reported the prevalence of prostate cancer in Sub-Saharan Africa, of which seven were from Nigeria (25, 28, 45, 64, 103, 116, 124), three from Malawi (67, 118, 133); consisting mainly of retrospective record review of patient files and registries as well as cross-sectional studies (116, 124). The two prevalence studies from Ghana consisted of a cross-sectional study (123) and a review (155). There was one study from each of the following countries: a retrospective record review of registry from Kenya (96), Uganda (59), a cross-sectional study from Cameroon (100), a retrospective record review of pathology reports from South Africa (80) and Zambia (44), a retrospective cohort study from Tanzania (49), a cross-sectional study from Rwanda (136). The two studies that reported on the whole of Africa (138, 154) and two global studies including Africa (104, 145).

##### Incidence Studies

A total of 17 studies focused on the incidence of prostate cancer in Sub-Saharan Africa. There was one systematic review covering the entire African region (150). There were two global studies (46, 117) and two SSA studies (87, 141). There were two retrospective record reviews of pathology reports (57) and registry (58) from Mozambique. There were also two retrospective record reviews from South Africa (36, 88) and Uganda (35, 78). There was one retrospective record review of pathology reports from Eritrea (60), Ethiopia (63), Malawi (37), one retrospective record review of patient files in Nigeria (29), one retrospective record review of registries from The Gambia (30) and Zimbabwe (38).

##### Other Burden Measures

A total of eight studies considered other measures of prostate cancer burden; mortality was measured by seven studies (33, 46, 117, 129, 135, 139, 144). DALYs were reported by three incidence studies (46, 117, 119). One study reported years of life lost (YLL) (135).

## Discussion

The objective of this scoping review was to map the evidence on the burden of the three most common cancers in Sub-Saharan Africa, namely cervical, breast and prostate cancers. We comprehensively searched PubMed, Web of Science and Scopus databases, and included 138 studies: 93 on cervical cancer, 67 on breast cancer, and 48 on prostate cancer. This shows a disproportionation in the distribution of cancer studies in SSA. Cervical and breast cancer receive more funding compared to prostate cancer, as they form sexual and reproductive health (SRH). Although breast cancer is the commonest cancer in SSA and globally (145), there are more studies on cervical cancer in the region. This could be because cervical cancer has been historically identified as AIDS-defining cancer (159), making cervical cancer screening a priority for women living with HIV. Poor resourced SSA countries have utilized the well-established HIV care platforms to screen for cervical cancer and donor funds have supported cervical cancer prevention and treatment programs in the region compared to breast cancer. While cervical cancer prevention HPV vaccines are widely available even in SSA, vaccinations against breast cancer are not yet available (160).

Majority of studies were done in South Africa and Nigeria. We identified some countries where there was no study on any of the three cancers. One of the reasons for this unavailability of cancer studies in some SSA countries could be because countries in SSA differ in terms of cancer control, with some countries putting in more resources than others. It has been reported that the cancer burden reported for SSA might be underestimated due to lack of appropriate screening, and diagnosis, poor access to treatment and care, and limitations in the technical workforce and infrastructure (14, 161). State-of-the-art cancer treatment and care, which includes cancer diagnostic equipment, is expensive, and since most countries in SSA are developing, they may not be able to acquire such equipment (162, 163). Additionally, the quality of the cancer data systems is poor, hence the possibility of having scarce research data in some of the SSA countries (164).

Most of the included studies were retrospective record review studies of mainly registries and patient files (*n* = 77), followed by cross-sectional studies (*n* = 51), in addition to some systematic reviews and prospective studies. The trend is similar elsewhere because cancer is a chronic condition which takes a while to detect; hence, most studies are done on existing data. Cross-sectional studies are also easier to perform since they involve cancer screening and surveillance activities that may be easily available at a public healthcare facility. There was only one study that included modeling and a few prospective studies were identified in this study. This shows that there is still a gap in research that employs modeling of the burden of prostate, cervical and breast cancer in SSA using prospective and statistical modeling. The main advantage of using modeling is that you estimate DALYs and the results can be standardized by age group, making it easier for interpretation. The benefits of using prospective studies include reporting of quality of life of the patients and assessing time-to events like mortality. These studies allow one to report results beyond prevalence and incidences and the risk of occurrence of an outcome of interest can be estimated, which is more informative than the prevalence estimate.

Majority of studies reported prevalence and incidence of the cancers, but we also found studies reporting mortality, DALYs, and YLL. The quality of studies was overall satisfactory. We also identified a gap in the literature on these three types of cancer in SSA. Firstly, there were countries with no specific study on any of these three types of cancer. Secondly, we found few nationwide population-based surveys of these cancers to determine their true prevalence since most studies reported the proportion of each of the cancers from oncology records where the target population were only cancer patients. Multi-country studies were relatively few in this study. To understand more about the regional picture of prostate, cervical and breast cancer in SSA, multi-country studies are more preferred as these studies show the heavily burdened countries across the region and those that are performing well. This comparison allows key stakeholders to share information and knowledge across the SSA region. Moreover, national surveys on cancer are important for monitoring and surveillance of non-communicable diseases like cancer indicators, to inform policy. The reduction in the burden of breast, prostate and cervical cancers in SSA starts with the implementation of efforts within each country; hence, nationwide studies are important and these can feed into multi-country studies.

To our knowledge, this is the first scoping review on the burden of cervical, breast and prostate cancers in SSA. There is a published scoping review (165) that mapped evidence on the prevalence, incidence, mortality and trends of HPV-associated cancers in SSA, which only found eight studies (six reviews and two quantitative studies). Another systematic review investigated only the incidence of prostate cancer in Africa (150). Our scoping review is different because we investigated the three most common cancers in SSA, namely the breast, cervical and prostate cancers. However, our conclusions in identifying gaps in effective cancer registry systems in Africa are similar to the previously published reviews.

We used a very experienced librarian who is also part of the co-authors to perform a comprehensive search in three main electronic databases. The search strategy used medical subject headings (MeSH) terms and was not limited in language or date; we searched all studies from inception to the current search date. Pairs of co-authors performed each of the processes, including screening of studies, data extraction, and charting, repeatedly. We used EndNote 20 software to manage the references and used the Rayyan software in screening the studies.

### Study Limitations

However, this study has some limitations. The main limitation is that we only searched three electronic databases and did not search the grey literature such as theses and dissertations of universities. We excluded some studies that did not report the cancer burden indicators defined in this scoping review. Nonetheless, since we identified 138 studies, it is more likely that these included studies are representative of the trends and patterns in the burden of cervical, breast and prostate cancer studies in SSA.

## Conclusions And Recommendations

We have identified a lack of comprehensive studies quantifying the burden of cervical, breast, and prostate cancers in SSA. This is crucial to help policy makers to decide on appropriate policies. Other countries in SSA do not have a single study on any of these three main cancers, thereby reflecting a great need for setting up research priorities, including funding frameworks for such essential studies. We recommend that countries in SSA expand their national surveys to include measures of cancer burden – more specifically the burden of cervical, breast and prostate cancers. Our scoping review enriched the pool of knowledge and provided a deeper understanding of the burden (incidence, prevalence, and DALYs) of cervical, breast, and prostate cancers in SSA, which could inform the public health systems' decision-making. Policy responses to the growing burden of cancer in general and more specifically that of cervical, breast, and prostate cancers will be required in less developed countries such as those in SSA to mitigate the threat caused by these cancers. This scoping review provided useful, relevant, timely and easily accessible information on the burden of the three cancers to facilitate the efforts by policymakers to decisively ramp up prevention and treatment interventions. These results can also help set research priorities for future cancer burden studies as well as help identify opportunities to improve existing policy frameworks around cancer prevention and treatment, especially for cervical, breast and prostate cancers in SSA.

## Author Contributions

AM conceptualized the topic with assistance from MMoy, MMoh, ZM-Z, HT, JB, GS, KK, NM, PN, PS, TE, and IM who contributed equally to the writing, research, critical review and proofreading of the manuscript. KK performed the search with assistance from MMoy and TE. All authors contributed to the article and approved the submitted version.

## Funding

This research was funded in part by the National Science Foundation, grant number 134651, to the MASAMU Advanced Study Institute.

## Conflict of Interest

The authors declare that the research was conducted in the absence of any commercial or financial relationships that could be construed as a potential conflict of interest.

## Publisher's Note

All claims expressed in this article are solely those of the authors and do not necessarily represent those of their affiliated organizations, or those of the publisher, the editors and the reviewers. Any product that may be evaluated in this article, or claim that may be made by its manufacturer, is not guaranteed or endorsed by the publisher.

## Abbreviations

MeSH: Medical Subject Headings
MMAT: Mixed Methods Appraisal Tool
PHC: Primary Healthcare
PRISMA-ScR: Preferred Reporting Items for Systematic Reviews and Meta-Analyses Extension for Scoping Reviews
UN: United Nations
WHO: World Health Organization.

## References

[1] Bray F Soerjomataram I. The changing global burden of cancer: transitions in human development and implications for cancer prevention and control. Cancer. (2015) 3:23–44. 10.1596/978-1-4648-0349-9_ch2 26913347

[2] Azubuike SO Muirhead C Hayes L McNally R. Rising global burden of breast cancer: the case of sub-Saharan Africa (with emphasis on Nigeria) and implications for regional development: a review. World J Surg Oncol. (2018) 16:63. 10.1186/s12957-018-1345-2 29566711 PMC5863808

[3] Popat K McQueen K Feeley TW. The global burden of cancer. Best Pract Res Clin Anaesthesiol. (2013) 27:399–408. 10.1016/j.bpa.2013.10.010 24267547

[4] Kanavos P. The rising burden of cancer in the developing world. Ann Oncol. (2006) 17 (Suppl 8):viii15-viii23. 10.1093/annonc/mdl983 16801335

[5] Ferlay J Soerjomataram I Dikshit R Eser S Mathers C Rebelo M. Cancer incidence and mortality worldwide: sources, methods and major patterns in GLOBOCAN 2012. Int J Cancer. (2015) 136:E359–86. 10.1002/ijc.29210 25220842

[6] Jedy-Agba E Joko WY Liu B Buziba NG Borok M Korir A. Trends in cervical cancer incidence in sub-Saharan Africa. Br J Cancer. (2020) 123:148–54. 10.1038/s41416-020-0831-9 32336751 PMC7341858

[7] International International agency for research on cancer (IARC) Cancer Tomorrow WHO 2018.

[8] Lott BE Trejo MJ Baum C McClelland DJ Adsul P Madhivanan P. Interventions to increase uptake of cervical screening in sub-Saharan Africa: a scoping review using the integrated behavioral model. BMC Public Health. (2020) 20:1–18. 10.1186/s12889-020-08777-4 32393218 PMC7216595

[9] Ntekim A. Cervical cancer in sub Sahara Africa. Topics Cer Cancer Advoc prevent. (2012) 4:54–9. 10.5772/2720026853214

[10] Somdyala NIM Bradshaw D Dhansay MA Stefan DC. Increasing Cervical Cancer Incidence in Rural Eastern Cape Province of South Africa From 1998 to 2012: A Population-Based Cancer Registry Study. JCO Glob Oncol. (2020) 6:1–8. 10.1200/JGO.19.00198 32031436 PMC7000228

[11] Sankaranarayanan R Budukh AM Rajkumar R. Effective screening programmes for cervical cancer in low- and middle-income developing countries. Bull World Health Organ. (2001) 79:954–62. 11693978 PMC2566667

[12] Habila MA Mantina N Kimaru LJ Musa J Ingram M Sagay A. Community engaged approaches to cervical cancer prevention and control in sub-saharan Africa: a scoping review protocol. Front Glob Women's Health. (2021) 2. 10.3389/fgwh.2021.697607 PMC8594022 34816234

[13] Arbyn M Weiderpass E Bruni L Sanjose Sde Saraiya M Ferlay J. Estimates of incidence and mortality of cervical cancer in 2018: a worldwide analysis. Lancet Glob Health. (2020) 8:e191–203. 10.1016/S2214-109X(19)30482-6 31812369 PMC7025157

[14] Odedina FT Ogunbiyi JO Ukoli FA. Roots of prostate cancer in African-American men. J Natl Med Assoc. (2006) 98:539–43. 16623066 PMC2569237

[15] R.I. Anorlu. Cervical cancer: the sub-Saharan African perspective. Reprod Health Matters. (2008) 16:41–9. 10.1016/S0968-8080(08)32415-X 19027621

[16] Jalloh M Niang L Ndoye M Labou I Gueye SM. Prostate cancer in Sub Saharan Africa. J Nephrol Urol Res. (2013) 1:15–20. 10.12970/2310-984X.2013.01.01.4

[17] Faridi R Zahra A Khan K Idrees M. Oncogenic potential of Human Papillomavirus (HPV) and its relation with cervical cancer. Virol J. (2011) 8:269. 10.1186/1743-422X-8-269 21635792 PMC3118362

[18] Ghebre RG Grover S Xu MJ Chuang LT Simonds H. Cervical cancer control in HIV-infected women: Past, present and future. Gynecol Oncol Rep. (2017) 21:101–8. 10.1016/j.gore.2017.07.009 28819634 PMC5548335

[19] Arksey H O'Malley L. Scoping studies: towards a methodological framework. Int J Soc Res Methodol. (2005) 8:19–32. 10.1080/1364557032000119616

[20] Levac D Colquhoun H O'Brien KK. Scoping studies: advancing the methodology. Implement Sci. (2010) 5:69. 10.1186/1748-5908-5-69 20854677 PMC2954944

[21] Peters MDJ Marnie C Tricco AC Pollock D Munn Z Alexander L. Updated methodological guidance for the conduct of scoping reviews. JBI Evid Synth. (2020) 18:2119–26. 10.11124/JBIES-20-00167 33038124

[22] Tricco AC Lillie E Zarin W O'Brien KK Colquhoun H Levac D. PRISMA Extension for Scoping Reviews (PRISMA-ScR): Checklist and Explanation. Ann Intern Med. (2018) 169:467–73. 10.7326/M18-0850 30178033

[23] Hong Q Pluye P Fàbregues S Bartlett G Boardman F Cargo M. Mixed Methods Appraisal Tool (MMAT) Version 2018: User Guide. Registration of Copyright 1148552 (2018) 34:285–91.

[24] Akarolo-Anthony SN Dal Maso L Igbinoba F Mbulaiteye SM Adebamowo CA. Cancer burden among HIV-positive persons in Nigeria: preliminary findings from the Nigerian AIDS-cancer match study. Infect Agents Cancer. (2014) 9:1. 10.1186/1750-9378-9-1 24597902 PMC3942812

[25] Anunobi CC Akinde OR Elesha SO Daramola AO Tijani KH Ojewola RW. Prostate diseases in Lagos, Nigeria: a histologic study with tPSA correlation. Niger Postgrad Med J. (2011) 18:98–104. 21670775

[26] Apffelstaedt JP Dalmayer L Baatjes K. Mammographic screening for breast cancer in a resource-restricted environment. South Afr Med J. (2014) 104:294–6. 10.7196/SAMJ.7246 25118556

[27] Arbyn M Castellsagué X Sanjosé Sde Bruni L Saraiya M Bray F. Worldwide burden of cervical cancer in 2008. Ann Oncol. (2011) 22:2675–86. 10.1093/annonc/mdr015 21471563

[28] Awodele O Adeyomoye AA Awodele DF Fayankinnu VB Dolapo DC. Cancer distribution pattern in south-western Nigeria. Tanzan J Health Res. (2011) 13:125–31. 10.4314/thrb.v13i2.55226 25566610

[29] Badmus TA Adesunkanmi AR Yusuf BM Oseni GO Eziyi AK Bakare TI. Burden of prostate cancer in southwestern Nigeria. Urology. (2010) 76:412–6. 10.1016/j.urology.2010.03.020 20451979

[30] Bah E Parkin DM Hall AJ Jack AD Whittle H. Cancer in the Gambia: 1988-97. Br J Cancer. (2001) 84:1207–14. 10.1054/bjoc.2001.1730 11336472 PMC2363873

[31] Balekouzou A Yin P Bekolo CE Pamatika CM Djeintote M Nambei SW. Histo-epidemiological profile of breast cancers among women in the Central African Republic: about 174 cases. BMC Cancer. (2018) 18:387. 10.1186/s12885-018-4256-2 29621999 PMC5887243

[32] Balekouzou A Yin P Pamatika CM Bishwajit G Nambei SW Djeintote M. Epidemiology of breast cancer: retrospective study in the Central African Republic. BMC Public Health. (2016) 16:1230. 10.1186/s12889-016-3863-6 27923361 PMC5142143

[33] Beksisa J Getinet T Tanie S Diribi J Hassen HY. Survival and prognostic determinants of prostate cancer patients in Tikur Anbessa Specialized Hospital, Addis Ababa, Ethiopia: a retrospective cohort study. PLoS ONE. (2020) 15:e0229854. 10.1371/journal.pone.0229854 32134996 PMC7058322

[34] Briggs ND Katchy KC. Pattern of primary gynecological malignancies as seen in a tertiary hospital situated in the Rivers State of Nigeria. Int J Gynaecol Obstet. (1990) 31:157–61. 10.1016/0020-7292(90)90714-V 1968863

[35] Bukirwa P Wabinga H Nambooze S Amulen PM Joko WY Liu BY. Trends in the incidence of cancer in Kampala, Uganda, 1991 to 2015. Int J Cancer. (2021) 148:2129–38. 10.1002/ijc.33373 33129228

[36] Cassim N Ahmad A Wadee R Rebbeck TR Glencross DK George JA. Prostate cancer age-standardised incidence increase between 2006 and 2016 in Gauteng Province, South Africa: A laboratory data-based analysis. South Afr Med J. (2021) 111:26–32. 10.7196/SAMJ.2020.v111i1.14850 33404002

[37] Chasimpha SJD Parkin DM Masamba L Dzamalala CP. Three-year cancer incidence in Blantyre, Malawi (2008-2010). Int J Cancer. (2017) 141:694–700. 10.1002/ijc.30777 28493322 PMC5999322

[38] Chokunonga E Borok MZ Chirenje ZM Nyakabau AM Parkin DM. Trends in the incidence of cancer in the black population of Harare, Zimbabwe 1991-2010. Int J Cancer. (2013) 133:721–9. 10.1002/ijc.28063 23364833

[39] Chokunonga E Levy LM Bassett MT Borok MZ Mauchaza BG Chirenje MZ. AIDS and cancer in Africa: The evolving epidemic in Zimbabwe. AIDS. (1999) 13:2583–8. 10.1097/00002030-199912240-00012 10630528

[40] Coghill AE Newcomb PA Madeleine MM Richardson BA Mutyaba I Okuku F. Contribution of HIV infection to mortality among cancer patients in Uganda. AIDS. (2013) 27:2933–42. 10.1097/01.aids.0000433236.55937.cb 23921614 PMC4319357

[41] DeGregorio GA Bradford LS Manga S Tih PM Wamai R Ogembo R. Prevalence, Predictors, and Same Day Treatment of Positive VIA Enhanced by Digital Cervicography and Histopathology Results in a Cervical Cancer Prevention Program in Cameroon. PLoS ONE. (2016) 11:e0157319. 10.1371/journal.pone.0157319 27280882 PMC4900564

[42] Dhokotera T Bohlius J Spoerri A Egger M Ncayiyana J Olago V. The burden of cancers associated with HIV in the South African public health sector, 2004-2014: A record linkage study. Infect Agents Cancer. (2019) 14:12. 10.1186/s13027-019-0228-7 31073325 PMC6500038

[43] Dykstra M Malone B Lekuntwane O Efstathiou J Letsatsi V Elmore S. Impact of Community-Based Clinical Breast Examinations in Botswana. JCO Glob Oncol. (2021) 7:17–26. 10.1200/GO.20.00231 33405960 PMC8081526

[44] Elem A Patil PS. Pattern of urological malignancy in Zambia. A hospital-based histopathological study. Br J Urol. (1991) 67:37–9. 10.1111/j.1464-410X.1991.tb15065.x 1847087

[45] Fapohunda A Fakolade A Omiye J Afolaranmi O Arowojolu O Oyebamiji T. Cancer presentation patterns in Lagos, Nigeria: Experience from a private cancer center. J Public Health Afr. (2020) 11:98–104. 10.4081/jphia.2020.1138 33623651 PMC7893315

[46] Fitzmaurice A Dicker D Pain A Hamavid H Moradi-Lakeh M Maclntyre MF. The Global Burden of Cancer 2013 Global Burden of Disease Cancer Collaboration. JAMA Oncol. (2015) 1:505–27. 10.1001/jamaoncol.2015.0735 26181261 PMC4500822

[47] Friebel-Klingner TM Luckett R Bazzett-Matabele L Ralefala TB Monare B. Clinical and sociodemographic factors associated with late stage cervical cancer diagnosis in Botswana. BMC Womens Health. (2021) 21:267. 10.1186/s12905-021-01402-5 34229672 PMC8259023

[48] Galukande M Wabinga H Mirembe F. Breast cancer survival experiences at a tertiary hospital in sub-Saharan Africa: a cohort study. World J Surg Oncol. (2015) 13:220. 10.1186/s12957-015-0632-4 26187151 PMC4506617

[49] Gunda A Kido I Kilonzo S Nkandala I Igenge J Mpondo B. Prevalence and Associated Factors of Incidentally Diagnosed Prostatic Carcinoma among Patients Who Had Transurethral Prostatectomy in Tanzania: A Retrospective Study. Ethiop J Health Sci. (2018) 28:11–8. 10.4314/ejhs.v28i1.3 29622903 PMC5866285

[50] Gyedu A Gaskill CE Agbedinu K Salazar DR Kingham TP. Surgical oncology at a major referral center in Ghana: Burden, staging, and outcomes. J Surg Oncol. (2018) 118:581–7. 10.1002/jso.25168 30095201 PMC6160332

[51] Hailu HE Mondul AM Rozek LS Geleta T. Descriptive Epidemiology of breast and gynecological cancers among patients attending Saint Paul's Hospital Millennium Medical College, Ethiopia. PLoS ONE. (2020) 15:e0230625. 10.1371/journal.pone.0230625 32196536 PMC7083302

[52] Horner MJ Chasimpha S Spoerri A Edwards J Bohlius J Tweya H. High Cancer Burden Among Antiretroviral Therapy Users in Malawi: A Record Linkage Study of Observational Human Immunodeficiency Virus Cohorts and Cancer Registry Data. Clin Infect Dis. (2019) 69:829–35. 10.1093/cid/ciy960 30452634 PMC6773978

[53] Horner MJ Salima A Chilima C Mukatipa M Kumwenda W Kampani C. Frequent HIV and Young Age Among Individuals With Diverse Cancers at a National Teaching Hospital in Malawi. J Glob Oncol. (2018) 4:1–11. 10.1200/JGO.17.00174 30085887 PMC6223526

[54] Ibrahim HM Ijaiya MA. Pattern of gynaecological malignancies at the University of Ilorin Teaching Hospital, Ilorin, Nigeria. J Obstet Gynaecol. (2013) 33:194–6. 10.3109/01443615.2012.738717 23445148

[55] Katz IT Butler LM Crankshaw TL Wright AA Bramhill K Leone DA. Cervical Abnormalities in South African Women Living With HIV With High Screening and Referral Rates. J Glob Oncol. (2016) 2:375–80. 10.1200/JGO.2015.002469 28717723 PMC5493244

[56] Kohler RE Tang J Gopal S Chinula L Hosseinipour MC Liomba NG. High rates of cervical cancer among HIV-infected women at a referral hospital in Malawi. Int J STD AIDS. (2016) 27:753–60. 10.1177/0956462415592999 26130691 PMC4870149

[57] Lorenzoni A Vilajeliu A Carrilho C Ismail MR Castillo P Augusto O. Trends in cancer incidence in Maputo, Mozambique, 1991-2008. PLoS ONE. (2015) 10:e0130469. 10.1371/journal.pone.0130469 26110774 PMC4481529

[58] Lorenzoni CF Ferro J Carrilho C Colombet M Parkin DM. Cancer in Mozambique: Results from two population-based cancer registries. Int J Cancer. (2020) 147:1629–37. 10.1002/ijc.32953 32142162

[59] Mbulaiteye SM Katabira ET Wabinga H Parkin DM Virgo P Ochai R. Spectrum of cancers among HIV-infected persons in Africa: The Uganda AIDS-Cancer Registry Match Study. Int J Cancer. (2006) 118:985–90. 10.1002/ijc.21443 16106415

[60] Medhin LB Achila OO Syum BE Gebremichael KH Said SM Lobeck H. Incidence of prostate cancer in Eritrea: Data from the National Health Laboratory, Orotta Referral Hospital and Sembel Hospital 2011-2018. PLoS ONE. (2020) 15:e0232091. 10.1371/journal.pone.0232091 32324838 PMC7179877

[61] Medhin LB Tekle LA Achila OO Said S. Incidence of Cervical, Ovarian and Uterine Cancer in Eritrea: Data from the National Health Laboratory, 2011-2017. Sci Rep. (2020) 10:9099. 10.1038/s41598-020-66096-5 32499531 PMC7272439

[62] Medhin LB Tekle LA Fikadu DT Sibhatu DB Gebreyohans SF Gebremichael KH. Incidence of Breast Cancer in Eritrea: a Retrospective Study from 2011 to 2017. Int J Breast Cancer. (2019) 2019:8536548. 10.1155/2019/8536548 31355003 PMC6633959

[63] Memirie ST Habtemariam MK Asefa M Deressa BT Abayneh G Tsegaye B. Estimates of Cancer Incidence in Ethiopia in 2015 Using Population-Based Registry Data. J Glob Oncol. (2018) 4:1–11. 10.1200/JGO.17.00175 30241262 PMC6223441

[64] Mohammed AZ Edino ST Ochicha O Gwarzo AK Samaila AA. Cancer in Nigeria: a 10-year analysis of the Kano cancer registry. Niger J Med. (2008) 17:280–4. 10.4314/njm.v17i3.37396 18788253

[65] Moodley M. Reduction in prevalence of invasive cervical cancer in KwaZulu-Natal, South Africa: impact of the human immunodeficiency virus epidemic. Int J Gynecol Cancer. (2006) 16:1036–40. 10.1111/j.1525-1438.2006.00588.x 16803482

[66] Moses A Mwafongo A Chikasema M Kafantenganji L Stanely C Chimzukira E. Risk factors for common cancers among patients at Kamuzu Central Hospital in Lilongwe, Malawi: a retrospective cohort study. Malawi Med J. (2017) 29:136–41. 10.4314/mmj.v29i2.11 28955421 PMC5610284

[67] Msyamboza KP Dzamalala C Mdokwe C Kamiza S Lemerani M Dzowela T. Burden of cancer in Malawi; common types, incidence and trends: national population-based cancer registry. BMC Res Notes. (2012) 5:149. 10.1186/1756-0500-5-149 22424105 PMC3327635

[68] Msyamboza KP Manda G Tembo B Thambo C Chitete L Mindiera C. Cancer survival in Malawi: a retrospective cohort study. Pan Afr Med J. (2014) 19:234. 10.11604/pamj.2014.19.234.4675 25838862 PMC4377240

[69] Mubarik S Yu Y Wang F Malik SS Liu X Fawad M. Epidemiological and sociodemographic transitions of female breast cancer incidence, death, case fatality and DALYs in 21 world regions and globally, from 1990 to 2017: An Age-Period-Cohort Analysis. J Adva Res. (2021) 37:185–96. 10.1016/j.jare.2021.07.012 35499053 PMC9039678

[70] Mungo C Cohen CR Maloba M Bukusi EA Huchko MJ. Prevalence, characteristics, and outcomes of HIV-positive women diagnosed with invasive cancer of the cervix in Kenya. Int J Gynaecol Obstet. (2013) 123:231–5. 10.1016/j.ijgo.2013.07.010 24095308 PMC4151462

[71] Nartey Y Hill PC Amo-Antwi K Nyarko KM Yarney J Cox B. Cervical Cancer in the Greater Accra and Ashanti Regions of Ghana. J Glob Oncol. (2017) 3:782–90. 10.1200/JGO.2016.005744 29244993 PMC5735962

[72] Nggada HA Yawe KDT Abdulazeez J Khalil MA. Breast cancer burden in Maiduguri, North eastern Nigeria. Breast J. (2008) 14:284–6. 10.1111/j.1524-4741.2008.00576.x 18476884

[73] Nkfusai NC Cumber SN Williams T Anchang-Kimbi JK Yankam BM Anye CS. Cervical cancer in the Bamenda Regional Hospital, North West Region of Cameroon: a retrospective study. Pan Afr Med J. (2019) 32, 90. 10.11604/pamj.2019.32.90.18217 31223381 PMC6560966

[74] Odida M Schmauz R Lwanga SK. Grade of malignancy of cervical cancer in regions of Uganda with varying malarial endemicity. Int J Cancer. (2002) 99:737–41. 10.1002/ijc.10384 12115509

[75] Odutola M Jedy-Agba EE Dareng EO Oga EA Igbinoba F Out T. Burden of cancers attributable to infectious agents in Nigeria: 2012-2014. Front Oncol. (2016) 6:216. 10.3389/fonc.2016.00216 27822455 PMC5075533

[76] Okonda S Wright C Michelow P. The status of cervical cytology in Swaziland, Southern Africa: a descriptive study. Cytojournal. (2009) 6:14. 10.4103/1742-6413.54916 19826481 PMC2758303

[77] Parham GP Mwanahamuntu MH Kapambwe S Muwonge R Bateman AC Blevins M. Population-level scale-up of cervical cancer prevention services in a low-resource setting: development, implementation, and evaluation of the cervical cancer prevention program in Zambia. PLoS ONE. (2015) 10:e0122169. 10.1371/journal.pone.0122169 25885821 PMC4401717

[78] Parkin DM Nambooze S Wabwire-Mangen F Wabinga HR. Changing cancer incidence in Kampala, Uganda, 1991-2006. Int J Cancer. (2010) 126:1187–95. 10.1002/ijc.24838 19688826

[79] Parkin DM Wabinga H Nambooze S Wabwire-Mangen F. AIDS-related cancers in Africa: maturation of the epidemic in Uganda. AIDS. (1999) 13:2563–70. 10.1097/00002030-199912240-00010 10630526

[80] Phiri-Ramongane B Khine A. Performance of free prostate-specific antigen ratio in differentiating between prostatic cancer and benign prostatic lesions at a referral hospital in South Africa. South Afr Fam Pract. (2018) 60:103–6. 10.4102/safp.v60i4.4889

[81] Rohner A Butikofer L Schmidlin K Sengayi M Maskew M Giddy J. Cervical cancer risk in women living with HIV across four continents: a multicohort study. Int J Cancer. (2020) 146:601–9. 10.1002/ijc.32260 31215037 PMC6898726

[82] Ruffieux Y Dhokotera T Muchengeti M Bartels L Olago V Bohlius J. Cancer risk in adolescents and young adults living with HIV in South Africa: a nationwide cohort study. Lancet HIV. (2021) 8:e614–22. 10.1016/S2352-3018(21)00158-2 34509198 PMC8491099

[83] Ruffieux Y Muchengeti M Egger M Efthimiou O Bartels L Olago V. Immunodeficiency and Cancer in 3.5 Million People Living With Human Immunodeficiency Virus (HIV): The South African HIV Cancer Match Study. Clin Infect Dis. (2021) 73:E735-E744. 10.1093/cid/ciab087 33530095 PMC8326558

[84] Saeed ME Cao J Fadul B Kadioglu O Khalid HE Yassin Z. A five-year survey of cancer prevalence in Sudan. Anticancer Res. (2016) 36:279–86. 26722054

[85] Sighoko D Kamaté B Traore C Mallé B Coulibaly B Karidiatou A. Breast cancer in pre-menopausal women in West Africa: analysis of temporal trends and evaluation of risk factors associated with reproductive life. Breast. (2013) 22:828–35. 10.1016/j.breast.2013.02.011 23489760

[86] Sitas A. Histologically diagnosed cancers in South Africa, 1988. South Afr Med J. (1994) 84:344–8. 7740381

[87] Soerjomataram I Lortet-Tieulent J Parkin DM Ferlay J Mathers C Forman D. Global burden of cancer in 2008: a systematic analysis of disability-adjusted life-years in 12 world regions. Lancet. (2012) 380:1840–50. 10.1016/S0140-6736(12)60919-2 23079588

[88] Somdyala NIM Bradshaw D Gelderblom WCA Parkin DM. Cancer incidence in a rural population of South Africa, 1998-2002. Int J Cancer. (2010) 127:2420–9. 10.1002/ijc.25246 20162610

[89] Somdyala NIM Parkin DM Sithole N Bradshaw D. Trends in cancer incidence in rural Eastern Cape Province; South Africa, 1998-2012. Int J Cancer. (2015) 136:E470–4. 10.1002/ijc.29224 25236502

[90] Ssemmanda S Katagirya E Bukirwa P Alele D Lukande R Kalungi S. Breast diseases histologically diagnosed at a tertiary facility in Uganda (2005-2014). BMC Cancer. (2018) 18:1285. 10.1186/s12885-018-5208-6 30577784 PMC6303921

[91] Tafadzwa D Julien R Lina B Eliane R Frederique C Leigh J. Spatiotemporal modelling and mapping of cervical cancer incidence among HIV positive women in South Africa: a nationwide study. Int J Health Geograph. (2021) 20:30. 10.1186/s12942-021-00283-z 34187465 PMC8244168

[92] Thomas JO Ojemakinde KO Ajayi IO Omigbodun AO Fawole OI Oladepo O. Population-based prevalence of abnormal cervical cytology findings and local risk factors in Ibadan, Nigeria: implications for cervical cancer control programs and human papilloma virus immunization. Acta Cytol. (2012) 56:251–8. 10.1159/000337444 22555526

[93] Umezulike AC Tabansi SN Ewunonu HA Nwana EJ. Epidemiological characteristics of carcinoma of the cervix in the Federal capital Territory of Nigeria. Niger J Clin Pract. (2007) 10:143–6. 17902507

[94] Wabinga HR Nambooze S Amulen PM Okello C Mbus L Parkin DM. Trends in the incidence of cancer in Kampala, Uganda 1991-2010. Int J Cancer. (2014) 135:432–9. 10.1002/ijc.28661 24615279

[95] Wabinga HR Parkin DM Wabwire-Mangen F Nambooze S. Trends in cancer incidence in Kyadondo County, Uganda, 1960-1997. Br J Cancer. (2000) 82:1585–92. 10.1054/bjoc.1999.1071 10789729 PMC2363394

[96] Wambalaba FW Son B Wambalaba AE. Nyong'o D, Nyong'o A. Prevalence and capacity of cancer diagnostics and treatment: a demand and supply survey of health-care facilities in Kenya. Cancer Control. (2019) 26:1073274819886930. 10.1177/1073274819886930 31795739 PMC6893940

[97] Wassie M Fentie B Asefa T. Determinants of Mortality among Cervical Cancer Patients Attending in Tikur Anbessa Specialized Hospital, Ethiopia: Institutional-Based Retrospective Study. J Oncol. (2021) 2021, 9916050. 10.1155/2021/9916050 34239565 PMC8233077

[98] Youlden DR Cramb SM Dunn NA Muller JM Pyke CM Baade PD. The descriptive epidemiology of female breast cancer: an international comparison of screening, incidence, survival and mortality. Cancer Epidemiol. (2012) 36:237–48. 10.1016/j.canep.2012.02.007 22459198

[99] Ali-Risasi C Verdonck K Padalko E Vanden Broeck D Praet M. Prevalence and risk factors for cancer of the uterine cervix among women living in Kinshasa, the Democratic Republic of the Congo: a cross-sectional study. Infect Agents Cancer. (2015) 10, 20. 10.1186/s13027-015-0015-z 26180542 PMC4502934

[100] Angwafo FF 3rd Zaher A Befidi-Mengue R Wonkam A Takougang I Powell I. High-grade intra-epithelial neoplasia and prostate cancer in Dibombari, Cameroon. Prostate Cancer Prostatic Dis. (2003) 6:34–8. 10.1038/sj.pcan.4500587 12664062

[101] Ayele W Addissie A Wienke A Unverzagt S Jemal A Taylor L. Breast awareness, self-reported abnormalities, and breast cancer in rural Ethiopia: a survey of 7,573 women and predictions of the national burden. Oncologist. (2021) 26:e1009–17. 10.1002/onco.13737 33650727 PMC8176994

[102] Bateman AC Katundu K Mwanahamuntu MH Kapambwe S Sahasrabuddhe VV Hicks ML. The burden of cervical pre-cancer and cancer in HIV positive women in Zambia: a modeling study. BMC Cancer. (2015) 15:541. 10.1186/s12885-015-1558-5 26205980 PMC4512016

[103] Bosland MC Nettey OS Phillips AA Anunobi CC Akinloye O Ekanem IA. Prevalence of prostate cancer at autopsy in Nigeria-A preliminary report. Prostate. (2021) 81:553–9. 10.1002/pros.24133 33905137

[104] Bray A Ren JS Masuyer E Ferlay J. Global estimates of cancer prevalence for 27 sites in the adult population in 2008. Int J Cancer. (2013) 132:1133–45. 10.1002/ijc.27711 22752881

[105] Chambuso R Ramesar R Kaambo E Murahwa AT Abdallah MOE De Sousa M. Age, absolute CD4 count, and CD4 percentage in relation to HPV infection and the stage of cervical disease in HIV-1-positive women. Medicine. (2020) 99:e19273. 10.1097/MD.0000000000019273 32118737 PMC7478573

[106] Curado M.P. Breast cancer in the world: incidence and mortality. Salud Publica Mex. (2011) 53:372–84. 22218791

[107] Dandena A Sinaga M Bezabih M. Patterns of breast fine neeedle aspiration cytoogy result among patients with breast complaints attending Jimma University specialized hospital, Southwestern Ethiopia. Ethiopian Med J. (2019) 57:259–64.

[108] Martel ADe Ferlay J Franceschi S Vignat J Bray F Forman D. Global burden of cancers attributable to infections in 2008: a review and synthetic analysis. Lancet Oncol. (2012) 13:607–15. 10.1016/S1470-2045(12)70137-7 22575588

[109] Martel ADe Plummer M Vignat J Franceschi S. Worldwide burden of cancer attributable to HPV by site, country and HPV type. Int J Cancer. (2017) 141:664–70. 10.1002/ijc.30716 28369882 PMC5520228

[110] Dhokotera T Bohlius J Egger M Spoerri A Ncayiyana JR Naidu G. Cancer in HIV-positive and HIV-negative adolescents and young adults in South Africa: a cross-sectional study. BMJ Open. (2021) 11:e043941. 10.1136/bmjopen-2020-043941 34663647 PMC8524277

[111] Donkoh ET Agyemang-Yeboah F Asmah RH Wiredu EK. Prevalence of cervical cancer and pre-cancerous lesions among unscreened Women in Kumasi, Ghana. Medicine (Baltimore). (2019) 98:e14600. 10.1097/MD.0000000000014600 30921178 PMC6456016

[112] Duncan ME Tibaux G Pelzer A Mehari L Peutherer J Young H. A socioeconomic, clinical and serological study in an African city of prostitutes and women still married to their first husband. Soc Sci Med. (1994) 39:323–33. 10.1016/0277-9536(94)90128-7 7939848

[113] Durowade KA Osagbemi GK Salaudeen AG Musa OI Akande TM Babatunde OA. Prevalence and risk factors of cervical cancer among women in an urban community of Kwara State, north central Nigeria. J Prev Med Hyg. (2012) 53:213–9. 23469591

[114] Elbasheer MMA Alkhidir AGA Mohammed SMA Abbas AAH Mohamed AO Bereir IM. Spatial distribution of breast cancer in Sudan 2010-2016. PLoS ONE. (2019) 14:e0211085. 10.1371/journal.pone.0211085 31525202 PMC6746353

[115] Etyang AO Munge K Bunyasi EW Matata L Ndila C Kapesa S. Burden of disease in adults admitted to hospital in a rural region of coastal Kenya: An analysis of data from linked clinical and demographic surveillance systems. Lancet Glob Health. (2014) 2:E216–24. 10.1016/S2214-109X(14)70023-3 24782954 PMC3986034

[116] Fatiregun O Sowunmi AC Habeebu M Okediji P Alabi A Fatiregun O. Prevalence and correlates of unmet supportive needs of nigerian patients with cancer. J Glob Oncol. (2019) 5:1–9. 10.1200/JGO.19.00043 31246552 PMC6613661

[117] Fitzmaurice C Akinyemiju TF Al Lami FH Alam T Alizadeh-Navaei R Allen C. Global, Regional, and National Cancer Incidence, Mortality, Years of Life Lost, Years Lived With Disability, and Disability-Adjusted Life-Years for 29 Cancer Groups, 1990 to 2016 A Systematic Analysis for the Global Burden of Disease Study. JAMA Oncol. (2018) 4:1553–68. 10.1200/JCO.2018.36.15_suppl.1568 29860482 PMC6248091

[118] Gopal S Krysiak R Liomba NG Horner MJ Shores CG Alide N. Early experience after developing a pathology laboratory in Malawi, with emphasis on cancer diagnoses. PLoS ONE. (2013) 8:e70361. 10.1371/journal.pone.0070361 23950924 PMC3737192

[119] Gouda HN Charlson F Sorsdahl K Ahmadzada S Ferrari AJ Erskine H. Burden of non-communicable diseases in sub-Saharan Africa, 1990-2017: results from the Global Burden of Disease Study 2017. Lancet Glob Health. (2019) 7:e1375–87. 10.1016/S2214-109X(19)30374-2 31537368

[120] Harding R Selman L Agupio G Dinat N Downing J Gwyther L. The prevalence and burden of symptoms amongst cancer patients attending palliative care in two African countries. Eur J Cancer. (2011) 47:51–6. 10.1016/j.ejca.2010.08.003 20822896

[121] He WQ Li C. Recent global burden of cervical cancer incidence and mortality, predictors, and temporal trends. Gynecol Oncol. (2021) 163:583–92. 10.1016/j.ygyno.2021.10.075 34688503

[122] Heer E Harper A Escandor N Sung H McCormack V Fidler-Benaoudia MM. Global burden and trends in premenopausal and postmenopausal breast cancer: a population-based study. Lancet Global Health. (2020) 8:e1027–37. 10.1016/S2214-109X(20)30215-1 32710860

[123] Hsing AW Yeboah E Biritwum R Tettey Y Marzo AMDe Adjei A. High prevalence of screen detected prostate cancer in West Africans: implications for racial disparity of prostate cancer. J Urol. (2014) 192:730–5. 10.1016/j.juro.2014.04.017 24747091 PMC4332806

[124] Ikuerowo SO Omisanjo OA Bioku MJ Ajala MO Mordi VP Esho JO. Prevalence and characteristics of prostate cancer among participants of a community-based screening in Nigeria using serum prostate specific antigen and digital rectal examination. Pan Afr Med J. (2013) 15:129. 10.11604/pamj.2013.15.129.2489 24255735 PMC3830465

[125] Joubert J Norman R Bradshaw D Goedecke JH Steyn NP Puoane T. Estimating the burden of disease attributable to excess body weight in South Africa in 2000. S Afr Med J. (2007) 97:683–90. 17952225

[126] Kapambwe S Sahasrabuddhe VV Blevins M Mwanahamuntu MH Mudenda V Shepherd BE. Age Distribution and Determinants of Invasive Cervical Cancer in a “Screen-and-Treat” Program Integrated With HIV/AIDS Care in Zambia. JAIDS-J Acquir Immune Defic Syndr. (2015) 70:E20–6. 10.1097/QAI.0000000000000685 26322673 PMC4791059

[127] Khalil AI Mpunga T Wei FX Baussano I de Martel C Bray F. Age-specific burden of cervical cancer associated with HIV: A global analysis with a focus on sub-Saharan Africa. Int J Cancer. (2021) 150:761–72. 10.1002/ijc.33841 34626498 PMC8732304

[128] La Ruche A Ramon R Mensah-Ado I Bergeron C Diomande M Sylla-Koko F. Squamous intraepithelial lesions of the cervix, invasive cervical carcinoma, and immunosuppression induced by human immunodeficiency virus in Africa. Dyscer-CI Group Cancer. (1998) 82:2401–8. 9635533

[129] Made F Wilson K Jina R Tlotleng N Jack S Ntlebi V. Distribution of cancer mortality rates by province in South Africa. Cancer Epidemiol. (2017) 51:56–61. 10.1016/j.canep.2017.10.007 29040965

[130] Makuza JD Nsanzimana S Muhimpundu MA Pace LE Ntaganira J Riedel DJ. Prevalence and risk factors for cervical cancer and pre-cancerous lesions in Rwanda. Pan Afr Med J. (2015) 22:26. 10.11604/pamj.2015.22.26.7116 26664527 PMC4662515

[131] McCormack VA Febvey-Combes O Ginsburg O Dos-Santos-Silva I. Breast cancer in women living with HIV: A first global estimate. Int J Cancer. (2018) 143:2732–40. 10.1002/ijc.31722 29992553

[132] Memiah P Makokha V Mbuthia W Kiiru GW Agbor S Odhiambo F. Epidemiology of Cervical Squamous Intraepithelial Lesions in HIV Infected Women in Kenya: a cross-Sectional Study. Afr J Reprod Health. (2015) 19:133–9. 26103703

[133] Mukhula V Sibale D Tarmahomed L Dzamalala C Msyamboza K Chasimpha S. Characterising cancer burden and quality of care at two palliative care clinics in Malawi. Malawi Med J. (2017) 29:130–5. 10.4314/mmj.v29i2.10 28955420 PMC5610283

[134] Naku Ghartey Jnr F Anyanful A Eliason S Mohammed Adamu S Debrah S. Pattern of Breast Cancer Distribution in Ghana: A Survey to Enhance Early Detection, Diagnosis, and Treatment. Int J Breast Cancer. (2016) 2016:3645308. 10.1155/2016/3645308 27635263 PMC5007313

[135] Nojilana B Bradshaw D Pillay-van Wyk V Msemburi W Laubscher R Somdyala NIM. Emerging trends in non-communicable disease mortality in South Africa, 1997 – 2010. South Afr Med J. (2016) 106:477–84. 10.7196/SAMJ.2016.v106i5.10674 27138667

[136] Nzeyimana A Nyirimodoka A Ngendahayo E Bonane A Muhawenimana E Umurangwa F. Diagnosis of advanced prostate cancer at the community level in Rwanda. Int Urol Nephrol. (2021) 53:1977–85. 10.1007/s11255-021-02921-8 34191229

[137] Ononogbu U Almujtaba M Modibbo F Lawal I Offiong R Olaniyan O. Cervical cancer risk factors among HIV-infected Nigerian women. BMC Public Health. (2013) 13:582. 10.1186/1471-2458-13-582 23767681 PMC3728111

[138] Parkin DM Bray F Ferlay J Jemal A. Cancer in Africa 2012. Cancer Epidemiol Biomark Preven. (2014) 23:953–66. 10.1158/1055-9965.EPI-14-0281 24700176

[139] Phillips-Howard PA Laserson KF Amek N Beynon CM Angell SY Khagayi S. Deaths Ascribed to Non-Communicable Diseases among Rural Kenyan Adults Are Proportionately Increasing: Evidence from a Health and Demographic Surveillance System, 2003-2010. PLoS ONE. (2014) 9:e114010. 10.1371/journal.pone.0114010 25426945 PMC4245262

[140] Pilleron S Sarfati D Janssen-Heijnen M Vignat J Ferlay J Bray F. Global cancer incidence in older adults, 2012 and 2035: a population-based study. Int J Cancer. (2019) 144:49–58. 10.1002/ijc.31664 29978474

[141] Pilleron S Soerjomataram I Charvat H Chokunonga E Somdyala NIM Wabinga H. Cancer incidence in older adults in selected regions of sub-Saharan Africa, 2008-2012. Int J Cancer. (2019) 144:1824–33. 10.1002/ijc.31880 30238972

[142] Ranganathan A Singh P Raghavendran K Wilkins EG Hamill JB Aliu O., The Global Macroeconomic Burden of Breast Cancer: Implications for Oncologic Surgery. Ann Surg. (2020) 274:1067–72. 10.1097/SLA.0000000000003662 32097168

[143] Sharma R. Breast cancer burden in Africa: evidence from GLOBOCAN 2018. J Public Health (Oxf). (2020) 43:763–71. 10.1093/pubmed/fdaa099 32657321

[144] Shibuya A Mathers CD Boschi-Pinto C Lopez AD Murray CJ. Global and regional estimates of cancer mortality and incidence by site: II. Results for the global burden of disease 2000. BMC Cancer. (2002) 2:37. 10.1186/1471-2407-2-37 12502432 PMC149364

[145] Sung H Ferlay J Siegel RL Laversanne M Soerjomataram I Jemal A. Global Cancer Statistics 2020: GLOBOCAN Estimates of Incidence and Mortality Worldwide for 36 Cancers in 185 Countries. CA Cancer J Clin. (2021) 71:209–49. 10.3322/caac.21660 33538338

[146] Tadele N. Evaluation of quality of life of adult cancer patients attending Tikur Anbessa specialized referral hospital, Addis Ababa Ethiopia. Ethiop J Health Sci. (2015) 25:53–62. 10.4314/ejhs.v25i1.8 25733785 PMC4337080

[147] Xi LF Toure P Critchlow CW Hawes SE Dembele B Sow PS. Prevalence of specific types of human papillomavirus and cervical squamous intraepithelial lesions in consecutive, previously unscreened, West-African women over 35 years of age. Int J Cancer. (2003) 103:803–9. 10.1002/ijc.10876 12516102

[148] Yang BH Bray FI Parkin DM Sellors JW Zhang ZF. Cervical cancer as a priority for prevention in different world regions: an evaluation using years of life lost. Int J Cancer. (2004) 109:418–24. 10.1002/ijc.11719 14961581 PMC4167424

[149] Zhang X Zeng Q Cai W Ruan W. Trends of cervical cancer at global, regional, and national level: data from the Global Burden of Disease study 2019. BMC Public Health. (2021) 21:894. 10.1186/s12889-021-10907-5 33975583 PMC8114503

[150] Adeloye D David RA Aderemi AV Iseolorunkanmi A Oyedokun A Iweala EE. An Estimate of the incidence of prostate cancer in africa: a systematic review and meta-analysis. PLoS ONE. (2016) 11:e0153496. 10.1371/journal.pone.0153496 27073921 PMC4830589

[151] Patel P Rose CE Collins PY Nuche-Berenguer B Sahasrabuddhe VV Peprah E. Noncommunicable diseases among HIV-infected persons in low-income and middle-income countries: a systematic review and meta-analysis. AIDS. (2018) 32:S5–s20. 10.1097/QAD.0000000000001888 29952786 PMC6380891

[152] Shrestha AD Neupane D Vedsted P Kallestrup P. Cervical cancer prevalence, incidence and mortality in low and middle income countries: a systematic review. Asian Pac J Cancer Prev. (2018) 19:319–24. 10.22034/APJCP.2018.19.2.319 29479954 PMC5980914

[153] Stelzle D Tanaka LF Lee KK Ibrahim Khalil A Baussano I Shah ASV. Estimates of the global burden of cervical cancer associated with HIV. Lancet Glob Health. (2021) 9:e161–9. 10.1016/S2214-109X(20)30459-9 33212031 PMC7815633

[154] Parkin DM Sitas F Chirenje M Stein L Abratt R Wabinga H. Part I: Cancer in indigenous Africans-Burden, distribution, and trends. Lancet Oncol. (2008) 9:683–92. 10.1016/S1470-2045(08)70175-X 18598933

[155] Yeboah E.D. Prevalence of benign prostatic hyperplasia and prostate cancer in Africans and Africans in the diaspora. J West Afr Coll Surg. (2016) 6:1–30. 29181363 PMC5667727

[156] Dano D Hénon C Sarr O Ka K Ba M Badiane A. Quality of Life During Chemotherapy for Breast Cancer in a West African Population in Dakar, Senegal: A Prospective Study. J Glob Oncol. (2019) 5:1–9. 10.1200/JGO.19.00106 31322991 PMC6690633

[157] Eber-Schulz P Tariku W Reibold C Addissie A Wickenhauser C Fathke C. Survival of breast cancer patients in rural Ethiopia. Breast Cancer Res Treat. (2018) 170:111–8. 10.1007/s10549-018-4724-z 29479644

[158] Coates MM Kintu A Gupta N Wroe EB Adler AJ Kwan GF. Burden of non-communicable diseases from infectious causes in 2017: a modelling study. Lancet Glob Health. (2020) 8:e1489–98. 10.1016/S2214-109X(20)30358-2 33098769 PMC8040338

[159] Finocchario-Kessler S Wexler C Maloba M Mabachi N Ndikum-Moffor F Bukusi E. Cervical cancer prevention and treatment research in Africa: a systematic review from a public health perspective. BMC Womens Health. (2016) 16:29. 10.1186/s12905-016-0306-6 27259656 PMC4893293

[160] Zachariah NN Basu A Gautam N Ramamoorthi G Kodumudi KN Kumar NB. Intercepting Premalignant, Preinvasive Breast Lesions Through Vaccination. Front Immunol. (2021) 12:4864. 10.3389/fimmu.2021.786286 34899753 PMC8652247

[161] Odedina FT Akinremi TO Chinegwundoh F Roberts R Yu D Reams RR. Prostate cancer disparities in Black men of African descent: a comparative literature review of prostate cancer burden among Black men in the United States, Caribbean, United Kingdom, and West Africa. Infect Agent Cancer. (2009) 4:1–8. 10.1186/1750-9378-4-S1-S2 19208207 PMC2638461

[162] Ukoli F Osime U Akereyeni F Okunzuwa O Kittles R Adams-Campbell L. Prevalence of elevated serum prostate-specific antigen in rural Nigeria. Int J Urol. (2003) 10:315–22. 10.1046/j.1442-2042.2003.00633.x 12757603

[163] Hamdi Y Abdeljaoued-Tej I Zatchi AA Abdelhak S Boubaker S Brown JS. Cancer in Africa: The Untold Story. Front Oncol. (2021) 11:650117. 10.3389/fonc.2021.650117 33937056 PMC8082106

[164] Ogunbiyi JO Shittu OB. Increased incidence of prostate cancer in Nigerians. J Natl Med Assoc. (1999) 91:159–64. 10203918 PMC2608450

[165] Lekoane KMB Kuupiel D Mashamba-Thompson TP Ginindza TG. Evidence on the prevalence, incidence, mortality and trends of human papilloma virus-associated cancers in sub-Saharan Africa: systematic scoping review. BMC Cancer. (2019) 19:563. 10.1186/s12885-019-5781-3 31185951 PMC6558783

