# Supplementary material for: Mapping Evidence on the Burden of Breast, Cervical, and Prostate Cancers in Sub-Saharan Africa: A Scoping Review
Source: Front Public Health. 2022 Jun 16;10:908302. doi: 10.3389/fpubh.2022.908302 (PMC9246362; doi:10.3389/fpubh.2022.908302)
Supplement: Supplementary file 1 [file Table_1.DOCX]

Supplementary Material

# Supplementary Table

**Database Search Strategy and Results for studies on the burden of cervical, breast and prostate cancer in Sub-Saharan Africa**

| Date | Database | Keywords | Number of retrieved studies |
| --- | --- | --- | --- |
| 18 November 2021 | Pubmed | (((("prostatic neoplasms"[MeSH Terms] OR Prostate cancer[Text Word] OR prostatic neoplasm*[Text Word] OR prostate tumour*[Text Word]) OR ("uterine cervical neoplasms"[MeSH Terms] OR Cervical cancer[Text Word] OR Cervix Cancer[Text Word] OR uterine cervical neoplasms[Text Word] OR cervical neoplasms[Text Word])) OR ("breast neoplasms"[MeSH Terms] OR Breast Cancer[Text Word] OR breast neoplasm[Text Word] OR Breast Tumor*[Text Word])) AND ("Global Burden of Disease"[MeSH Terms] OR burden[Text Word] OR "prevalence"[MeSH Terms] OR prevalence[Text Word])) AND (("africa south of the sahara"[MeSH Terms] OR Sub saharan Africa[Text Word] OR SSA[Text Word] OR Sub-saharan Africa[Text Word] OR Sub sahara Africa[Text Word]) OR (("Africa"[MeSH Terms] OR AFRICA[Text Word]) NOT ("Africa, Northern"[Mesh] OR Northern Africa[Text Word]))) | 1056 |
| 18 November 2021 | Scopus | ( TITLE-ABS-KEY ( "prostat* neoplasm" OR "Prostat* cancer" OR "prostat* tumo*r" OR "Cancer of the Prostate" OR "uterine cervical neoplasm*" OR "Cervical cancer" OR "Cervix Cancer" OR "Cancer of the cervix" OR "breast neoplasms" OR "Breast Cancer*" OR "breast carcinoma" OR "cancer of the breast" ) AND TITLE-ABS-KEY ( "Global Burden of Disease" OR "Disease Global Burden*" OR "Burden of Disease" OR burden OR prevalence* ) AND TITLE-ABS-KEY ( "sub saharan africa" OR "sub-saharan africa" OR "sub sahara" OR sub-sahara OR ssa OR africa ) AND NOT TITLE-ABS-KEY ( algeria OR egypt OR libya OR morocco OR tunisia ) ) | 765 |
| 18 November 2021 | Web of Science | (((ALL=(“prostat* neoplasm” OR “Prostat* cancer” OR “prostat* tumo*r” OR “Cancer of the Prostate” OR "uterine cervical neoplasm*" OR “Cervical cancer” OR “Cervix Cancer” OR “Cancer of the cervix” OR "breast neoplasms" OR “Breast Cancer*” OR “breast carcinoma” OR “cancer of the breast” )) AND ALL=("Global Burden of Disease" OR “Disease Global Burden*” OR “Burden of Disease" OR burden OR prevalence*)) AND ALL=(“sub saharan africa” or “sub-saharan africa” or “sub sahara” or sub-sahara or ssa or Africa)) NOT ALL=(algeria OR egypt OR libya OR morocco OR tunisia) | 930 |

**
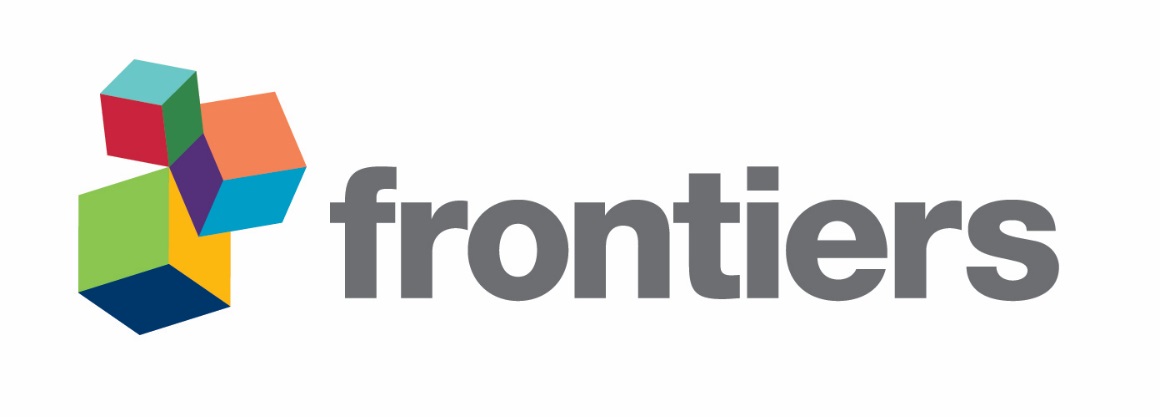
**
